# Short-Term Cooling Increases Plasma ANGPTL3 and ANGPTL8 in Young Healthy Lean Men but Not in Middle-Aged Men with Overweight and Prediabetes

**DOI:** 10.3390/jcm8081214

**Published:** 2019-08-14

**Authors:** Laura G.M. Janssen, Matti Jauhiainen, Vesa M. Olkkonen, P.A. Nidhina Haridas, Kimberly J. Nahon, Patrick C.N. Rensen, Mariëtte R. Boon

**Affiliations:** 1 Department of Medicine, Division of Endocrinology, Leiden University Medical Center, P.O. Box 9600, 2300 RC Leiden, The Netherlands; 2 Einthoven Laboratory for Experimental Vascular Medicine, Leiden University Medical Center, P.O. Box 9600, 2300 RC Leiden, The Netherlands; 3 Minerva Foundation Institute for Medical Research, Biomedicum 2U, 00290 Helsinki, Finland

**Keywords:** adipose tissue, ANGPTL3, ANGPTL4, ANGPTL8, lipid metabolism

## Abstract

Angiopoietin-like proteins (ANGPTLs) regulate triglyceride (TG)-rich lipoprotein distribution via inhibiting TG hydrolysis by lipoprotein lipase in metabolic tissues. Brown adipose tissue combusts TG-derived fatty acids to enhance thermogenesis during cold exposure. It has been shown that cold exposure regulates ANGPTL4, but its effects on ANGPTL3 and ANGPTL8 in humans have not been elucidated. We therefore investigated the effect of short-term cooling on plasma ANGPTL3 and ANGPTL8, besides ANGPTL4. Twenty-four young, healthy, lean men and 20 middle-aged men with overweight and prediabetes were subjected to 2 h of mild cooling just above their individual shivering threshold. Before and after short-term cooling, plasma ANGPTL3, ANGPTL4, and ANGPTL8 were determined by ELISA. In young, healthy, lean men, short-term cooling increased plasma ANGPTL3 (+16%, *p* < 0.05), ANGPTL4 (+15%, *p* < 0.05), and ANGPTL8 levels (+28%, *p* < 0.001). In middle-aged men with overweight and prediabetes, short-term cooling only significantly increased plasma ANGPTL4 levels (+15%, *p* < 0.05), but not ANGPTL3 (230 ± 9 vs. 251 ± 13 ng/mL, *p* = 0.051) or ANGPTL8 (2.2 ± 0.5 vs. 2.3 ± 0.5 μg/mL, *p* = 0.46). We show that short-term cooling increases plasma ANGPTL4 levels in men, regardless of age and metabolic status, but only overtly increases ANGPTL3 and ANGPTL8 levels in young, healthy, lean men.

## 1. Introduction

Increased plasma triacylglycerol (TG) levels are an independent risk factor for cardiovascular disease [1]. TG is either derived from dietary lipids or synthesized by the liver and white adipose tissue (WAT) from glucose and carried in the circulation within TG-rich lipoproteins (TRLs). TRLs can be hydrolysed by lipoprotein lipase (LPL) on endothelial cells to provide underlying oxidative tissues with fatty acids (FA) as fuel during increased energy demands (fasting or exercise) or to increase lipid storage in WAT during nutrient excess [2].

As energy needs can rapidly change, the LPL-mediated clearance of TRL-derived TG is under strict regulation of several factors including lipoprotein-associated apolipoproteins (e.g., APOC2 and APOC3) and angiopoietin-like proteins (ANGPTLs) [3]. ANGPTLs consist of a family of multifunctional glycoproteins, of which ANGPTL3, ANGPTL4, and ANGPTL8 inhibit LPL activity and work in concert to regulate lipoprotein metabolism [4]. Loss-of-function mutations in either one of these proteins are associated with a favourable lipid profile including lower TG levels in humans [5,6,7,8]. Moreover, deficiency for either one of these proteins in mouse models results in lower plasma TG levels, whereas overexpression leads to hypertriglyceridemia [9,10,11,12,13].

A recently identified novel player in TG metabolism is brown adipose tissue (BAT). Mouse studies have shown that activating BAT reduces plasma TG [14], mainly via LPL-mediated processing of TRLs [15] and alleviates dyslipidemia and atherosclerosis [16]. Therefore, BAT activation is currently widely investigated as potential treatment strategy aiming to improve cardiometabolic diseases in humans [17]. The main function of BAT is to generate heat by combustion of intracellular lipids to maintain core body temperature, and the LPL-dependent FA influx into activated brown adipocytes is required to replenish these intracellular lipid stores. The most potent physiological stimulus of BAT activation is cold exposure, which results in sympathetic activation of β-adrenergic receptors on brown adipocytes [18].

Cold exposure was previously shown to affect ANGPTL4 expression in adipose tissue in mice and to increase circulating ANGPTL4 levels in men [19,20]. The effects of cold exposure on ANGPTL3 and ANGPTL8 in humans have not been established as yet. We, therefore, investigated the effect of short-term cooling on plasma ANGPTL3 and ANGPTL8, in comparison with ANGPTL4, in relation to changes in lipid metabolism in young, healthy, lean men as well as middle-aged men with overweight and prediabetes.

## 2. Experimental Section

### 2.1. Study Design and Participants

In this study, blood samples of two clinical trials were used; one consisting of a cohort of young, healthy, lean men [21] (Dutch Trial Register 2473) and one consisting of a cohort of middle-aged men with overweight and prediabetes [22] (Clinicaltrials.gov NCT02291458). Both studies were approved by the Medical Ethical Committee of the LUMC and conducted in accordance with the principles of the revised Declaration of Helsinki (2013) and the Medical Research Involving Human Subjects Act (WMO). All subjects signed written informed consent prior to participation.

The study setup of the young, healthy, lean cohort was described in detail elsewhere [21]. In short, the study investigated the effect of cold exposure on brown adipose tissue and energy metabolism in 24 healthy, lean (BMI < 25 kg/m^2^) men, aged 18–28 years, of white Caucasian (*n* = 12) and South Asian (*n* = 12) descent. Subjects were included between March 2013 and June 2013. After an overnight fast, body fat percentage was measured by dual-energy X-ray absorptiometry (iDXA, GE Healthcare, UK). A blood sample was collected at thermoneutrality and at the end of an individualized water-cooling protocol, and here analyzed for plasma ANGPTL3, ANGPTL4, ANGPTL8, and serum lipids. The individualized cooling protocol consisted of approximately 2 h mild cooling between 2 water-perfused blankets; 1 above and 1 beneath the study subject (Blanketrol III, Cincinnati Sub-Zero Products, Cincinnati, OH, USA). The individualized cooling protocol started at 32 °C and the water temperature was gradually decreased until shivering occurred, after which the temperature was increased by 3–4 °C to stop shivering. Hereafter, an [^18^F]FDG-PET/CT scan was performed to quantify BAT volume and glucose uptake.

An elaborate description of the study setup of the middle-aged overweight prediabetic cohort can be found elsewhere [22]. Briefly, 20 middle-aged (40–55 years) white Caucasian (*n* = 10) and South Asian (*n* = 10) men with overweight or obesity (BMI 25–35 kg/m^2^) and prediabetes were included in a randomized double-blind cross-over study, evaluating the effect of *L*-arginine on brown adipose tissue and energy metabolism. Subjects were included between October 2014 and June 2015. Prediabetes was defined according to ADA criteria as having either fasting plasma glucose levels between 5.6–6.9 mmol/L or plasma glucose levels 2 h after an oral glucose tolerance test between 7.8–11.1 mmol/L [23]. Two South Asian subjects used simvastatin 40 mg once daily. While subjects received either L-arginine or placebo for 6 weeks, in the current study only data after ingestion of the placebo were used. Placebo tablets consisted of a mixture of pregelatinized maize starch, microcrystalline cellulose, and magnesium stearate. Placebo supplements were divided over 3 gifts: after breakfast, lunch, and dinner. On the following day after placebo intake, after 4 h of fasting in the morning, a blood sample was collected under thermoneutral conditions. Hereafter, an individualized mild cooling protocol lasting approximately 2 h was initiated, after which another blood sample was collected. The individualized cooling protocol consisted of gradually lowering the water temperature until just above the shivering point of the subjects, here being wrapped in a water-perfused suit (ThermaWrap Universal 3166, MTRE Advanced Technologies, Yavne, Israel). In the current study, we assessed these blood samples for serum lipids and plasma ANGPTL3, ANGPTL4, and ANGPTL8 as well as plasma glucose and insulin. The following day after the cooling experiment, body fat percentage was determined with DXA (Discovery A, Hologic, Bedford, MA, USA).

In both the young, healthy, lean cohort and middle-aged overweight prediabetic cohort, subjects were instructed not to exercise more than 3 times per week and to refrain from exercise prior to the experimental day. In both study cohorts, subjects were also instructed to not change their dietary habits and to consume a standardized evening meal prior to the experimental day.

### 2.2. Serum and Plasma Analyses

Plasma samples of both the young, healthy, and lean and middle-aged overweight prediabetic cohorts were analysed for ANGPTL3 [24] and ANGPTL8 [25] by in-house developed ELISAs. For the ANGPTL8 ELISA, in brief, antibodies against multiple synthesized ANGPTL8 peptides were chosen from different parts of the ANGPTL8 protein molecule. Rabbit R355 antibodies against the ANGPTL8 amino acid region 54–68 were combined with the horseradish peroxidase-labelled capture antibody against the ANGPTL8 peptide region 182–196 (rabbit R360) [25]. Plasma samples were also analysed for ANGPTL4 with a commercial ELISA assay (R&D Systems, Minneapolis, MN, USA). The intra- and inter-assay coefficients of variation for ANGPTL3 and ANGPTL4 were <15% [24]. Precision or intra- and inter-assay CVs for ANGPTL8 were approximately 10%. Serum TG and free fatty acid (FFA) concentrations were determined with enzymatic kits in both the young, healthy, lean cohort (Roche Diagnostics, Woerden, the Netherlands and Wako Chemicals, Neuss, Germany, respectively) and the middle-aged overweight prediabetic cohort (ABX Pentra 400 autoanalyzer, HORIBA Medical, Montpellier, France). In the middle-aged overweight prediabetic cohort, plasma glucose was also measured with an enzymatic kit (ABX Pentra 400 autoanalyzer, HORIBA Medical, Montpellier, France) and plasma insulin via a commercially available radioimmunoassay kit (Human Insulin-specific Radioimmunoassay, Millipore Corporation, Burlington, MA, USA). Four young, healthy, lean subjects and 1 middle-aged subject with overweight and prediabetes were excluded from this study due to absent plasma samples.

### 2.3. Statistical Analysis

Statistical analyses were performed with SPSS Statistics version 25 for Windows (IBM, Armonk, NY, USA). Baseline characteristics were compared between cohorts and ethnicities with a two-tailed unpaired student’s *t*-test, and with a two-way mixed-effect ANOVA in case temperature was an additional factor. A linear mixed-model analysis was performed with cohort, ethnicity, and temperature modelled as fixed effects, to investigate the effect of cold exposure on ANGPTLs both within and between cohorts and ethnicities. Temperature was additionally modelled as a random effect with intercepts and an unstructured covariance type. Correlations between changes in plasma ANGPTLs and serum lipids were performed using linear regression analysis and were assessed for interaction of ethnicity. Data are presented as mean ± SEM, unless stated otherwise. A *p*-value < 0.05 was considered statistically significant. No correction for multiple testing was applied.

## 3. Results

### 3.1. Clinical Characteristics

Clinical characteristics of the healthy lean cohort as well as effects of short-term cooling on serum lipids and other metabolic parameters have been described elsewhere [21]. In brief, subjects were 24 ± 1 years of age, had a BMI of 21.9 ± 0.4 kg/m^2^, and a body fat percentage of 21.4 ± 1.2% (Table 1). Short-term cooling increased both serum TG (+0.22 ± 0.06 mmol/L, *p <* 0.01) and FFA levels (+0.19 ± 0.05 mmol/L, *p* < 0.001; Table 1). Similar observations were made when taking into account South Asian and white Caucasian ethnicities separately (Table S1).

Clinical characteristics for the middle-aged men with overweight and prediabetes have also been described in detail elsewhere [22]. Compared with the young, healthy, lean men, middle-aged overweight prediabetic subjects were older (47 ± 2 vs. 24 ± 1 years, *p* < 0.001), had a higher BMI (30.6 ± 0.8 vs. 21.9 ± 0.4 kg/m^2^, *p* < 0.001) and a higher body fat percentage (30.9 ± 0.9 vs. 21.4 ± 1.2%, *p* < 0.001) (Table 1). In addition, compared with the young, healthy, lean men, FFA levels were lower (0.54 ± 0.04 vs. 0.84 ± 0.08 mmol/L, *p* < 0.01) and TG levels higher (1.56 ± 0.14 vs. 0.87 ± 0.10 mmol/L, *p* < 0.001) in middle-aged overweight prediabetic subjects (Table 1). Short-term cooling increased serum TG (+0.18 ± 0.04 mmol/L, *p* < 0.001) but not FFA levels (+0.06 ± 0.04 mmol/L, *p* = 0.11) in middle-aged men with overweight and prediabetes. Similar observations were made when taking into account South Asian and white Caucasian ethnicities separately (Table S1).

### 3.2. Short-Term Cooling Increases Plasma ANGPTL3 and ANGPTL8 in Young, Healthy, Lean Men but Not in Middle-Aged Men with Overweight and Prediabetes

**Figure 1:**
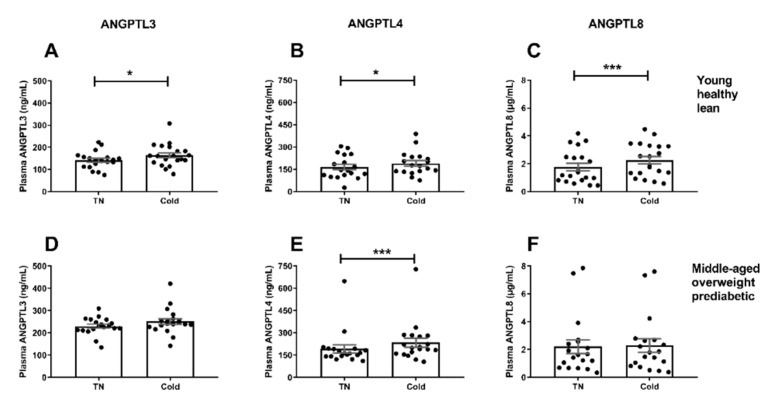
Effect of cold exposure on plasma ANGPTL3, ANGPTL4, and ANGPTL8 levels in young, healthy, lean men (**A**–**C**) and middle-aged men with overweight and prediabetes (**D**–**F**). Data are mean ± SEM. *** *p* < 0.001, * *p* < 0.05 cold vs. thermoneutrality (TN).

In young, healthy, lean men, short-term cooling increased ANGPTL3 (142 ± 9 vs. 164 ± 11 ng/mL, +16%, *p* < 0.05; Figure 1A), ANGPTL4 (165 ± 19 vs. 190 ± 19 ng/mL, +15%, *p* < 0.05; Figure 1B), and ANGPTL8 levels (1.8 ± 0.3 vs. 2.3 ± 0.3 μg/mL, +28%, *p* < 0.001; Figure 1C).

In middle-aged men with overweight and prediabetes, short-term cooling also increased ANGPTL4 levels (193 ± 27 vs. 234 ± 31 ng/mL, +15%, *p* < 0.001; Figure 1E). In contrary to the young, healthy, lean men, short-term cooling did not overtly increase ANGPTL3 (230 ± 9 vs. 251 ± 13 ng/mL, *p* = 0.051; Figure 1D) or ANGPTL8 levels (2.2 ± 0.5 vs. 2.3 ± 0.5 μg/mL, *p* = 0.46; Figure 1F). The effect of cold on plasma ANGPTL8 levels was significantly different between the young, healthy, lean men and the middle-aged men with overweight and prediabetes (+28% vs. +3%, *p* < 0.01). Similar trends were observed for cold-induced ANGPTL4 and ANGPTL8 levels in both cohorts when taking into account South Asian and white Caucasian men separately. However, in case of ANGPTL3, plasma levels increased in white Caucasian but not South Asian middle-aged men with overweight and prediabetes (Figure S1).

### 3.3. The Change in Plasma ANGPTL4 Negatively Correlates with the Change in Triglycerides after Short-Term Cooling in Young, Healthy, Lean Men

To further investigate whether the change in ANGPTLs during short-term cooling was related to the increase in serum lipids, we performed correlation analyses between the changes in the ANGPTLs and TG and FFA levels in both study cohorts.

**Figure 2:**
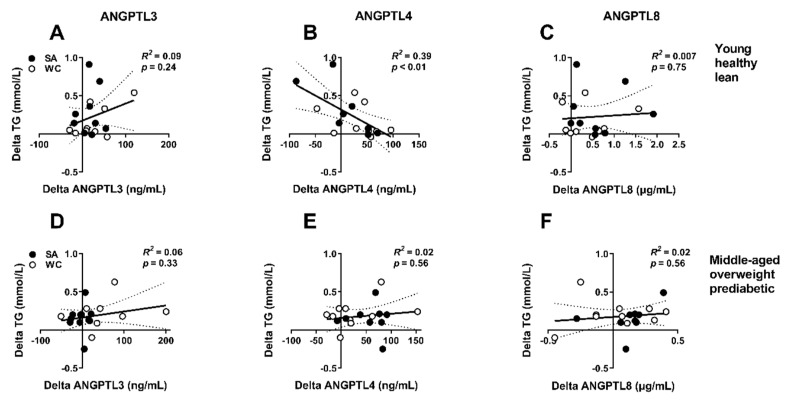
Correlation between cold-induced changes in serum triglyceride (TG) and plasma ANGPTL3, ANGPTL4, and ANGPTL8 levels in young, healthy, lean men (**A**–**C**) and middle-aged men with overweight and prediabetes (**D**–**F**). Dotted lines represent 95% confidence interval. TG = triglycerides. Black circles are South Asians (SA), white circles are white Caucasians (WC).

Data of both ethnicities were pooled, as ethnic origin did not show interaction with any of the correlation analyses. We did not observe a correlation between the cold-induced response in either one of the ANGPTLs and FFA levels in the young, healthy, lean men or middle-aged men with overweight and prediabetes. In the young, healthy, lean men, the cold-induced response in ANGPTL4 negatively correlated with the cold-induced response in TG (*R*^2^ = 0.39, *p* < 0.01; Figure 2B), whereas no correlations were observed for ANGPTL3 (Figure 2A) or ANGPTL8 (Figure 2C) levels. In addition, no correlations between the cold-induced response in ANGPTL3, ANGPTL4, and ANGPTL8 and TG levels were observed in the middle-aged men with overweight and prediabetes (Figure 2D–F). Of note, body fat percentage did not correlate with cold-induced changes in levels of either of the ANGPTLs (Figure S2). Moreover, body fat percentage did not affect any of the correlation analyses between cold-induced changes in ANGPTLs and TG or FFA levels.

### 3.4. Changes in ANGPTLs are not Overtly Correlated to [^18^F]FDG Uptake by BAT or Plasma Glucose or Insulin Levels after Short-Term Cooling

Short-term cooling not only increases TRL-derived FA uptake, but also glucose uptake to stimulate thermogenesis by BAT, the latter likely to enhance de novo lipogenesis [26]. ANGPTLs have a well-established role in TRL-derived FA uptake by metabolic tissues, but their interplay with glucose metabolism is more controversial [27]. We therefore evaluated cold-induced changes in levels of ANGPTLs in relation to [^18^F]FDG uptake by BAT on PET/CT scan in both young, healthy, lean men and middle-aged men with overweight and prediabetes. We additionally assessed cold-induced changes in ANGPTL levels in relation to delta glucose, delta insulin levels, as well as HOMA-IR under insulin resistant conditions (i.e., in the cohort of middle-aged men with overweight and prediabetes).

Data of both ethnicities were pooled, as ethnic origin did not show interaction with any of the correlation analyses aside from ANGPTL8. We did not observe correlations between the cold-induced response in either one of the ANGPTLs and BAT volume, SUVmean, or BAT metabolic activity (BAT volume multiplied by SUVmean) in the young, healthy, lean men (Figure S3). In the middle-aged men with overweight and prediabetes, we only observed a negative correlation between the cold-induced change in ANGPTL4 levels and BAT volume, but no correlations between other ANGPTLs and BAT parameters (Figure S4). In addition, we did not observe correlations between the cold-induced response in either one of the ANGPTLs and delta glucose, delta insulin levels, or HOMA-IR in the middle-aged men with overweight and prediabetes (Figure S5).

## 4. Discussion

ANGPTLs are inhibitors of LPL activity and function in modulating TRLs that traffic between tissues depending on specific situational energy demands. During cold exposure, LPL-mediated hydrolysis of TRL-TG by BAT is enhanced to meet the increased FA demand to facilitate thermogenesis [15]. Here, we confirmed that short-term cooling increases plasma ANGPTL4 levels in both young, healthy, lean men and middle-aged men with overweight and prediabetes. In addition, we now show that cooling increases plasma ANGPTL3 and ANGPTL8 levels, but only in young, healthy, lean men. We propose that the elevated circulating ANGPTL3 and ANGPTL8 levels during short-term cooling represent a compensatory response aimed at preventing an ANGPTL4-promoted excessive lipid accumulation in oxidative tissues in young, healthy, lean men.

First, we show that short-term cooling increased plasma ANGPTL4 levels in both young, healthy, lean men and middle-aged men with overweight and prediabetes. We previously obtained serum ANGPTL4 levels in this same young, healthy, lean cohort, and these findings are in line albeit measured with a different ELISA [20]. Plasma ANGPTL4 levels also increased after 48 h of continuous mild cold exposure (16 °C) in young obese males [19]. In addition to cold exposure, both fasting and exercise increased circulating ANGPTL4 levels in humans [28,29,30]. Regulation of *Angptl4* expression is tissue-specific, as fasting upregulated *Angptl4* expression and impaired LPL activity in WAT in mice, thereby facilitating the uptake of TG-derived FA by tissues with an increased energy demand [28,31]. In line with this, in mice, cold exposure upregulated *Angptl4* expression and limited TRL-derived FA uptake in WAT, whereas *Angptl4* expression was downregulated and, as a consequence, TG-derived FA uptake was increased in BAT [19,31]. We previously hypothesized that during short-term cooling in humans, FAs derived from intracellular lipolysis bind to peroxisome proliferator-activated receptor-γ to stimulate ANGPTL4 expression in WAT, thereby increasing circulating ANGPTL4 levels [32]. This may subsequently limit TG-derived FA uptake by WAT and redirect TRLs towards active BAT for hydrolysis of TG [20]. In the current study, we also observed a negative correlation between the cold-induced changes in ANGPTL4 levels and TG levels in young, healthy, lean men. According to our hypothesis, a higher increase in plasma ANGPTL4 levels during short-term cooling possibly indicates more shuttling of TRLs away from WAT towards active BAT, which might be accompanied by enhanced TRL-TG hydrolysis by BAT that subsequently results in a less pronounced cold-induced increase in serum TG.

In addition to ANGPTL4, we now show that short-term cooling increased plasma ANGPTL3 levels in young, healthy, lean men. ANGPTL3 in humans is nearly exclusively expressed in the liver and inhibits LPL activity in metabolic tissues in an endocrine fashion [33,34]. In contrast to ANGPTL4, ANGPTL3 inhibits LPL activity and TG-derived FA uptake by oxidative tissues after (re)feeding, thereby promoting lipid storage in WAT. This was demonstrated by a study showing that *Angptl3^-/-^* mice are unable to suppress LPL activity specifically in oxidative tissues in a fed state, thereby increasing very-low density-lipoprotein (VLDL)-TG-derived FA uptake by oxidative tissues (skeletal muscle, heart, and BAT) and reducing VLDL-TG-derived FA uptake by WAT. As a consequence, plasma TG levels were markedly lower in *Angptl3^-/-^* mice compared with wild-type mice [35]. In addition to TG levels, plasma FFA and glycerol levels were lower in *Angptl3^-/-^* mice, likely due to impaired inhibition of lipolysis [36]. Lowering circulating TG levels via ANGPTL3 inactivation by antisense oligonucleotides or monoclonal antibodies is a promising treatment strategy to target dyslipidemia and cardiovascular disease, as this reduced atherosclerosis progression in mice and significantly improved lipid profile in subjects with dyslipidemia in phase I trials [7,37]. However, the effect of pharmacologically targeting ANGPTL3 on risk factors for cardiovascular disease in specific metabolically challenged subjects remains to be elucidated in future studies.

We also show that short-term cooling increases plasma ANGPTL8 levels in young, healthy, lean men. This is in line with mouse studies showing that cold exposure enhances expression of *Angptl8* in liver, BAT, and WAT, although circulating ANGPTL8 levels were not reported in these studies [31,38]. The expression of ANGPTL8 is enriched in liver and present to a lesser extent in WAT and BAT and is highly upregulated in both tissues after (re)feeding [12,39]. Similar to ANGPTL3, ANGPTL8 likely inhibits LPL activity and TG-derived FA uptake by oxidative tissues after feeding to promote lipid storage in WAT. This was evident from a study showing that *Angptl8^-/-^* mice have increased LPL activity only in oxidative tissues (heart and skeletal muscle) and not in WAT upon (re)feeding [11]. Moreover, Wang et al. [13] showed that *Angptl8^-/-^* mice have impaired uptake of VLDL-TG-derived FA by WAT in a fed state.

ANGPTL3 and ANGPTL8 share sequence homology, form a protein–protein complex, and need each other’s presence to sufficiently regulate circulating lipid levels [12]. Zhang et al. [40] proposed the idea of an ANGPTL3-4-8 axis that ensures adequate distribution of TRL-TG to energy-demanding tissues in different nutritional states. In this model, during fasting, ANGPTL4 negatively regulates LPL activity in WAT to redirect TRL-TG towards other energy requiring tissues for hydrolysis, whereas upon feeding, ANGPTL3 and ANGPTL8 negatively regulate LPL activity in oxidative tissues to make TG-derived FA available for storage by WAT. In this context, we hypothesize that the increase in plasma ANGPTL4 during short-term cooling indicates inhibition of LPL activity in WAT to shuttle TRLs towards active BAT for uptake of TG-derived FA, whereas ANGPTL3 and ANGPTL8 redirect TRLs towards WAT to prevent the accumulation of excess lipids in active BAT in young, healthy, lean men.

It is tempting to speculate about the underlying mechanisms that increase plasma ANGPTL3 and ANGPTL8 during short-term cooling in young, healthy, lean men. Both ANGPTL3 and ANGPTL8 are regulated by the liver X receptor (LXR) [41,42,43]. Oxysterols are metabolites of cholesterol and are endogenous ligands of the LXR. As mouse studies have shown that cold exposure rapidly generates TRL-derived cholesterol-enriched remnants that are cleared by the liver [16], this might provide a source of cholesterol that enhances oxysterol formation and thereby stimulates LXR-induced expression of ANGPTL3 and ANGPTL8. Possibly, reduced formation of oxysterols as a consequence of decreased hepatic clearance of cholesterol-enriched remnants under insulin resistant conditions is involved in the absent response of ANGPTL3 and ANGPTL8 during short-term cooling in our cohort of middle-aged men with overweight and prediabetes [44]. Besides activation of the LXR, ANGPTL8 expression is upregulated in both hepatocytes and adipocytes by insulin [34,39,45]. As insulin release is stimulated during cold exposure [46], we speculate that insulin might contribute to the increased plasma ANGPTL8 levels during short-term cooling. Similar to other metabolic tissues, BAT becomes less sensitive to insulin during (pre)diabetes [47]. Therefore, we speculate that the absent cold-induced changes in ANGPTL3 and ANGPTL8 in middle-aged men with overweight and prediabetes might reflect an attempt of the body to overcome impaired glucose uptake by insulin-resistant BAT. It should be noted that we did not observe correlations between cold-induced changes in ANGPTL3 or ANGPTL8 and changes in glucose or insulin levels to support these hypotheses. However, circulating levels of insulin may not reflect local signaling function in metabolic tissues, including its effects on ANGPTL8 secretion. We also did not observe overt correlations between cold-induced changes in ANGPTLs and [^18^F]FDG uptake by BAT measured with PET/CT scan. However, this only reflects the uptake of glucose by BAT. Taking into account the LPL-inhibitory function of ANGPTLs, it would be highly interesting to specifically investigate cold-induced changes in ANGPTLs in relation to the uptake of (TRL-derived) FA by BAT in future studies. Of note, we observed an increase in FFA levels upon short-term cooling in the young, healthy, lean cohort but not in the middle-aged overweight prediabetic cohort. Interestingly, an increased fat mass is associated with an impaired FFA release from subcutaneous WAT [48]. This likely reflects impaired lipolysis, which may be mediated in part via catecholamine-resistance during obesity (reviewed in [49]). We therefore propose that reduced activity of the sympathetic nervous system in our cohort of overweight and obese men contributed to their unchanged FFA levels upon short-term cooling.

A strong aspect of our study is that we evaluated the effects of short-term cooling on ANGPTLs in both a cohort of healthy and metabolically challenged men. However, this study also had its limitations. For example, its cross-sectional design, as it would be interesting to investigate whether differences in cold-induced ANGPTL levels arise during metabolic changes within individuals over time. In addition, the study designs of both cohorts, although comparable to a certain extent, are not identical with respect to timing and fasting duration (which may explain lower baseline FFA levels in the middle-aged vs. young cohort). As the applied cooling protocols are also slightly different, we cannot exclude that variability in temperatures using individualized cooling protocols affected ANGPTL and lipid levels. Additionally, it is likely that a fasting state, maintained during the short-term cooling, partly contributed to an increase in circulating lipids and ANGPTL4, whereas this might have abolished the increase in ANGPTL3 and ANGPTL8. Importantly, we cannot exclude possible confounding in our analyses by a difference in age between both study cohorts, nor an effect of the placebo in the middle-aged overweight prediabetic cohort. Lastly, as the sample size of both studies is small, larger studies are warranted to confirm these observations.

## 5. Conclusions

In conclusion, we show that short-term cooling not only increases plasma ANGPTL4, but also plasma ANGPTL3 and ANGPTL8 levels in young, healthy, lean men. We propose that these ANGPTLs act in concert to facilitate TG partitioning between tissues in response to cold. While ANGPTL4 likely functions to shuttle TRLs away from WAT towards active thermogenic tissues during cold exposure, we suggest that ANGPTL3 and ANGPTL8 redirect TRLs away from thermogenic tissues to prevent excessive lipid accumulation. Whether these increases in circulating ANGPTLs reflect their ability to locally inhibit LPL-mediated TG hydrolysis and subsequent TG-derived FA uptake by metabolic tissues, remains to be elucidated.

## Figures and Tables

**Table 1:** Clinical characteristics. Data are mean ± SEM. *** *p* < 0.001, ** *p* < 0.01 middle-aged overweight prediabetic men vs. young, healthy, lean men. BMI = body mass index, FFA = free fatty acids, TG = triglycerides. Four healthy, young, lean subjects and one middle-aged overweight subject were excluded from the original cohorts due to absent plasma samples.

*Clinical Characteristics*	Young Healthy Lean Men (*n* = 20)	Middle-Aged Overweight Prediabetic Men (*n* = 19)
Age (years)	24 ± 1	47 ± 2 ***
Height (m)	1.79 ± 0.02	1.78 ± 0.01
Weight (kg)	70.6 ± 2.1	96.9 ± 2.9 ***
BMI (kg/m^2^)	21.9 ± 0.4	30.6 ± 0.8 ***
Body fat percentage	21.4 ± 1.2	30.9 ± 0.9 ***
Thermoneutral TG (mmol/L)	0.87 ± 0.10	1.56 ± 0.14 ***
Cold-induced change TG (mmol/L)	+0.22 ± 0.06	+0.18 ± 0.04
Thermoneutral FFA (mmol/L)	0.84 ± 0.08	0.54 ± 0.04 **
Cold-induced change FFA (mmol/L)	+0.19 ± 0.05	+0.06 ± 0.04 ^p = 0.053^

## Supplementary Materials

The following are available online at https://www.mdpi.com/2077-0383/8/8/1214/s1, Figure S1: Effect of cold exposure on plasma ANGPTL3, ANGPTL4 and ANGPTL8 levels in young healthy lean South Asian and white Caucasian men, and in South Asian and white Caucasian middle-aged men with overweight and prediabetes; Figure S2: Correlation between body fat percentage and cold-induced changes in plasma ANGPTL3, ANGPTL4 and ANGPTL8 levels in the young healthy lean and middle-aged overweight prediabetic cohorts; Figure S3: Correlation between cold-induced changes in plasma ANGPTL3, ANGPTL4 and ANGPTL8 levels and BAT volume, SUVmean and metabolic activity, i.e., volume*SUVmean, in young healthy lean men; Figure S4: Correlation between cold-induced changes in plasma ANGPTL3, ANGPTL4 and ANGPTL8 levels and BAT volume, SUVmean and metabolic activity, i.e., volume*SUVmean, in middle-aged men with overweight and prediabetes; Figure S5: Correlation between cold-induced changes in plasma ANGPTL3, ANGPTL4 and ANGPTL8 levels and cold-induced changes in plasma glucose and insulin levels and HOMA1-IR in middle-aged men with overweight and prediabetes; Table S1: Clinical characteristics per ethnicity.
