# Supplementary material for: Short-Term Cooling Increases Plasma ANGPTL3 and ANGPTL8 in Young Healthy Lean Men but Not in Middle-Aged Men with Overweight and Prediabetes
Source: J Clin Med. 2019 Aug 14;8(8):1214. doi: 10.3390/jcm8081214 (PMC6723119; doi:10.3390/jcm8081214)
Supplement: Supplementary file 1 [file jcm-08-01214-s001.pdf]

Clinical characteristics	Young healthy lean men		Middle-aged overweight prediabetic men	
	SA (N=10)	WC (N=10)	SA (N=9)	WC (N=10)
Age (years)	24±1	25±1	46±3 ***	48±2 ***
Height (m)	1.75±0.02 ††	1.84±0.01	1.75±0.02 †	1.81±0.02
Weight (kg)	65.6±3.0†	75.6±2.2	93.5±4.2 ***	99.9±4.0 ***
BMI (kg/m^2)	21.4±0.7	22.3±0.4	30.4±1.1 ***	30.7±1.2 ***
Body fat percentage	23.4±1.6	19.4±1.5	31.6±1.4 **	30.1±1.0 ***
Thermoneutral TG (mmol/L)	0.91±0.18	0.82±0.08	1.58±0.26 *	1.54±0.16 **
Cold-induced change TG (mmol/L)	+0.29±0.11	+0.16±0.07	+0.15±0.06	+0.21±0.06
Thermoneutral FFA (mmol/L)	0.97±0.13	0.71±0.10	0.59±0.04 *	0.49±0.07
Cold-induced change FFA (mmol/L)	+0.04±0.06 ††	+0.34±0.05	+0.01±0.04	+0.11±0.06 **

Table S1. Clinical characteristics per ethnicity. Data are mean ± SEM. ***$p < 0.001$, **$p < 0.01$, *$p < 0.05$ within same ethnicity between study cohorts. ††$p < 0.01$, †$p < 0.05$ between ethnicities within same study cohort. BMI = body mass index, FFA = free fatty acids, SA = South Asian, TG = triglycerides, WC = white Caucasian. Four healthy young lean subjects and 1 middle-aged overweight subject were excluded from the original cohorts due to absent plasma samples.

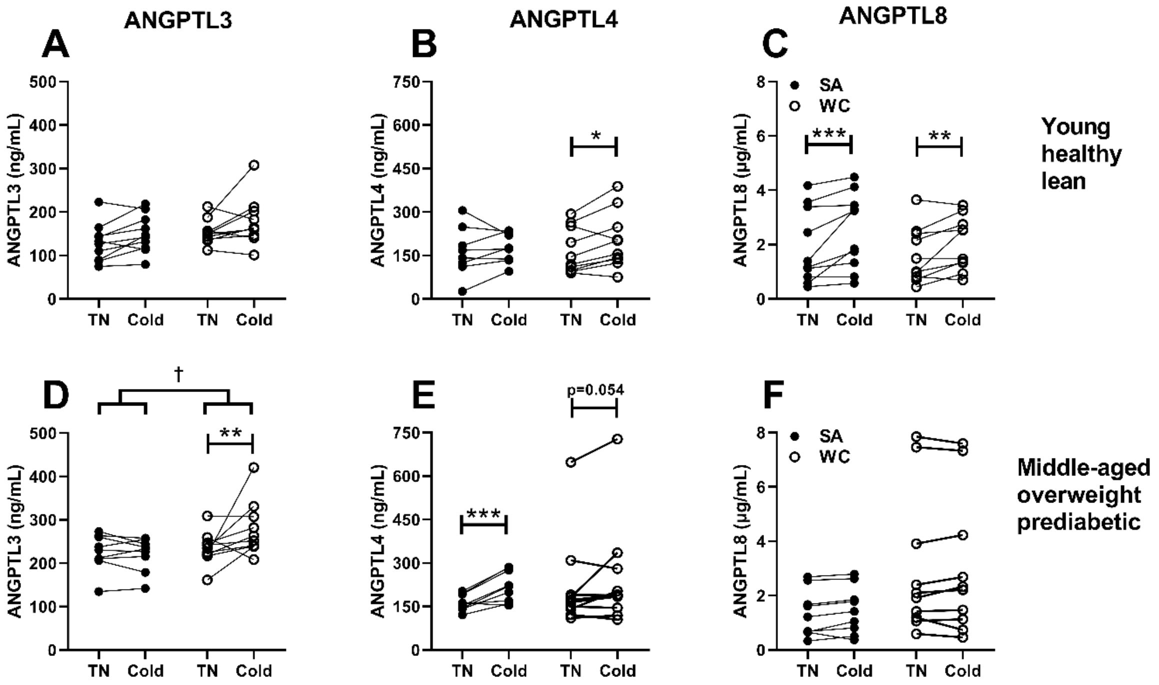

Figure S1. Effect of cold exposure on plasma ANGPTL3, ANGPTL4 and ANGPTL8 levels in young healthy lean South Asian (SA) and white Caucasian (WC) men **(A-C)** and SA and WC middle-aged men with overweight and prediabetes **(D-F)**. Data are mean. ***$p < 0.001$, **$p < 0.01$, *$p < 0.05$ cold *vs* thermoneutrality (TN). †$p < 0.05$ cold-induced delta SA *vs* WC. Black circles are SA, white circles are WC.

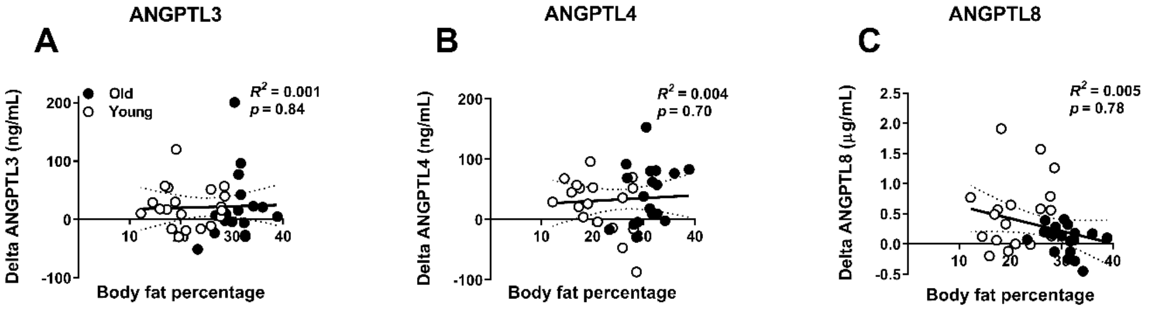

Figure S2. Correlation between body fat percentage and cold-induced changes in plasma ANGPTL3 (**A**), ANGPTL4 (**B**) and ANGPTL8 levels (**C**) in the young healthy lean and middle-aged overweight prediabetic cohorts combined. Dotted lines represent 95% confidence interval. Black circles are the middle-aged overweight prediabetic subjects (old) and white circles are the young healthy lean subjects (young).

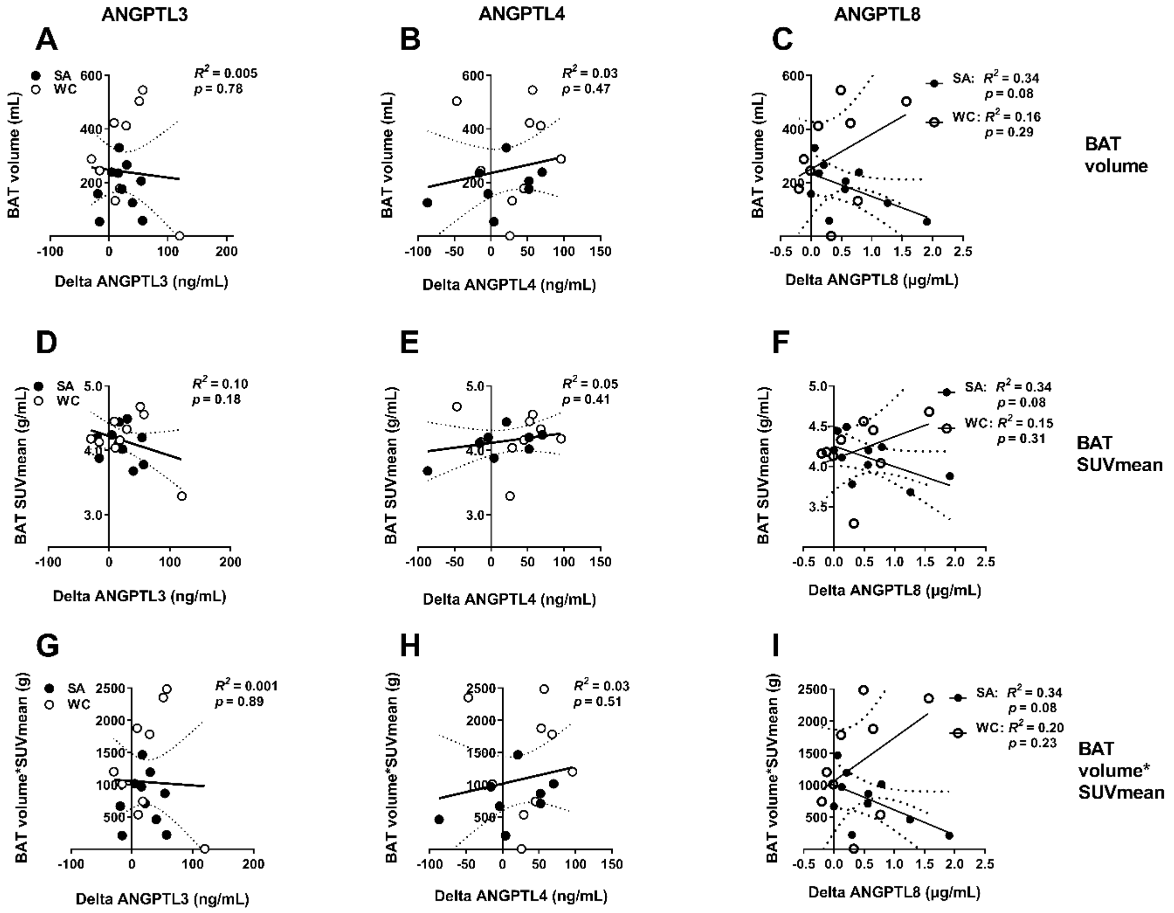

Figure S3. Correlation between cold-induced changes in plasma ANGPTL3, ANGPTL4 and ANGPTL8 levels and BAT volume (**A-C**), SUVmean (**D-F**) and metabolic activity, i.e., volume*SUVmean, (**G-I**) in young healthy lean men. Dotted lines represent 95% confidence interval. Black circles are South Asians (SA), white circles are white Caucasians (WC).

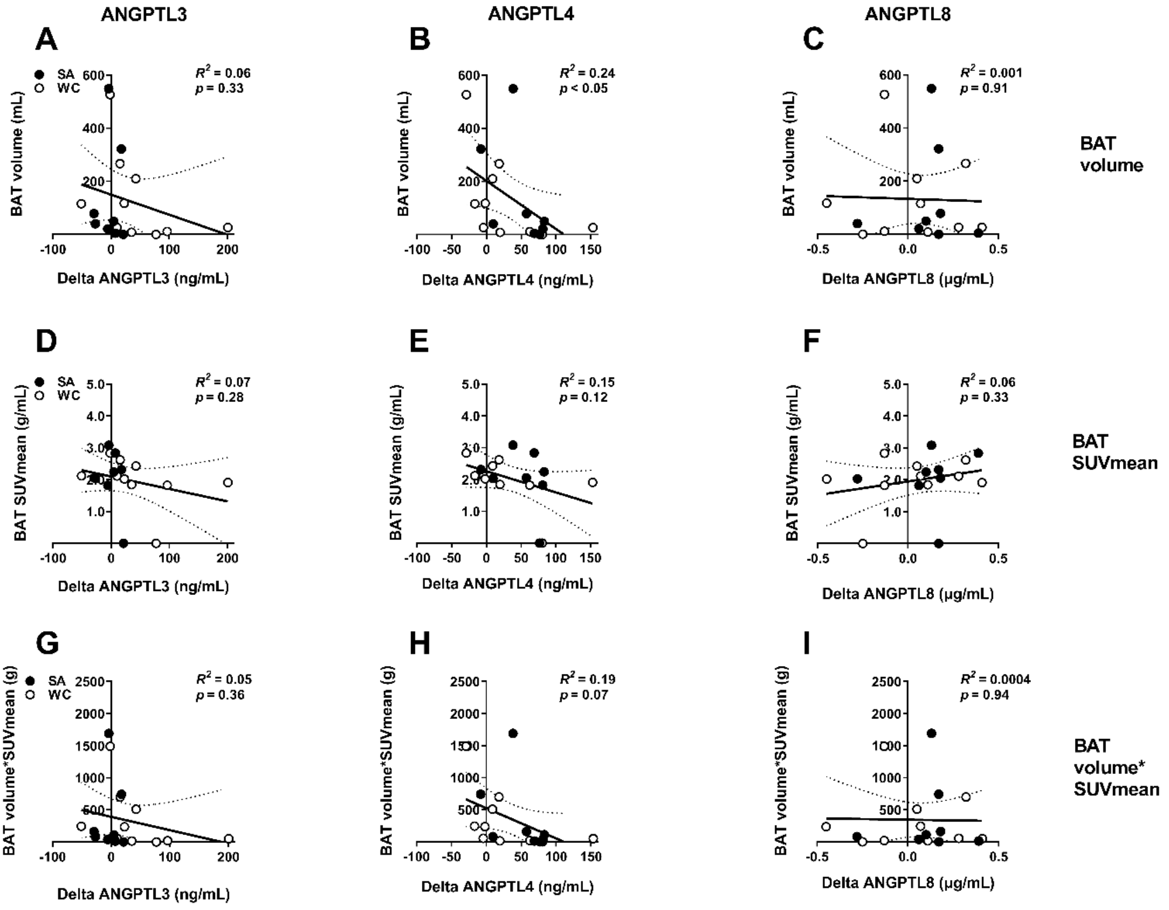

Figure S4. Correlation between cold-induced changes in plasma ANGPTL3, ANGPTL4 and ANGPTL8 levels and BAT volume (**A-C**), SUVmean (**D-F**) and metabolic activity, i.e., volume*SUVmean, (**G-I**) in middle-aged men with overweight and prediabetes. Dotted lines represent 95% confidence interval. Black circles are South Asians (SA), white circles are white Caucasians (WC).

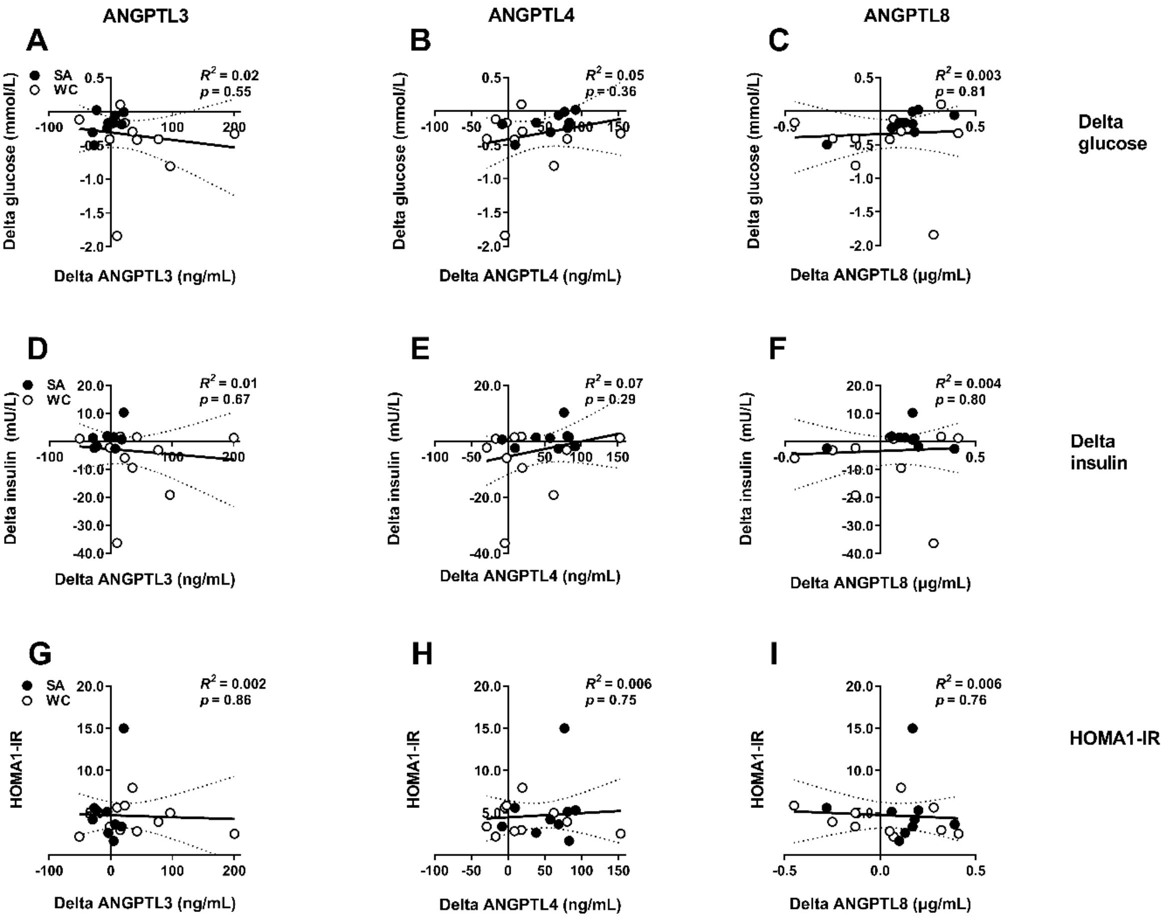

Figure S5. Correlation between cold-induced changes in plasma ANGPTL3, ANGPTL4 and ANGPTL8 levels and cold-induced changes in plasma glucose (**A-C**) and insulin (**D-F**) levels and HOMA1-IR (**G-H**) in middle-aged men with overweight and prediabetes. Dotted lines represent 95% confidence interval. Black circles are South Asians (SA), white circles are white Caucasians (WC).
